# Endoscopic Over Under Cartilage Tympanoplasty Is Not Inferior to Underlay Cartilage Tympanoplasty

**DOI:** 10.1097/ONO.0000000000000005

**Published:** 2021-11-05

**Authors:** Isaac D. Erbele, Madelinn R. Fink, Gauri Mankekar, Leslie S. Son, Moisés A. Arriaga, Rahul Mehta

**Affiliations:** 1 Division of Neurotology, Department of Otolaryngology, Louisiana State University Health Sciences Center, Baton Rouge and New Orleans, Louisiana; 2 Department of Otolaryngology, Brooke Army Medical Center, Fort Sam Houston, Texas; 3 Department of Surgery, Uniformed Services University of the Health Sciences, Bethesda, Maryland; 4 Louisiana State University Health Sciences Center, New Orleans, Louisiana; 5 Department of Otolaryngology, Louisiana State University Health Sciences Center Shreveport, Shreveport, Louisiana.

**Keywords:** Complications, Hearing, Tympanic membrane perforation, Tympanoplasty

## Abstract

**Objective:**

Evaluate whether elevating the tympanic membrane from the malleus during endoscopic tympanoplasty may negatively affect postoperative hearing outcomes or perforation rates by comparing 2 similar endoscopic tympanoplasty techniques.

**Study Design:**

Retrospective cohort.

**Setting:**

Tertiary care center.

**Patients:**

Endoscopic over-under cartilage tympanoplasties age and gender matched to endoscopic underlay cartilage tympanoplasties between January 2015 and January 2019. Exclusion criteria included preoperative or intraoperative cholesteatoma, performance of mastoidectomy or ossicular chain reconstruction, and lack of postoperative audiogram.

**Interventions:**

Endoscopic cartilage tympanoplasty via over-under or underlay technique.

**Main Outcome Measures:**

Pre- and postoperative pure-tone average and word recognition score, graft success.

**Results:**

A total of 52 patients were evaluated: 26 endoscopic over-under cartilage tympanoplasties were matched to endoscopic underlay cartilage tympanoplasties. Both groups demonstrated a statistically significant improvement in air conduction hearing (9 dB [*P* < 0.001] and 6 dB [*P* < 0.01], respectively), and bone pure-tone average did not worsen in either group (*P* < 0.001 and *P* < 0.05, respectively). Postoperative air conduction pure-tone average was statistically noninferior in the over-under group compared with the underlay group (*P* < 0.05). Reperforation was present in 3 patients (12%) in the underlay group and none in the over-under group, but this difference was not statistically significant (*P* = 0.24).

**Conclusions:**

Endoscopic over-under cartilage tympanoplasty effectively closes tympanic membrane perforations and improves hearing, without greater risk than underlay tympanoplasty. Elevating the tympanic membrane from the malleus does not confer worsen hearing outcomes.

A tympanoplasty is a surgical procedure to repair tympanic membrane perforations or atelectasis, as well as an adjunct to more extensive middle ear and mastoid procedures. Lifting the tympanic membrane from the malleus can be a helpful technique to assess the entire mesotympanum, as well as to repair otherwise inaccessible perforations of the anterior superior quadrant.(1–4) This may explain why some prominent endoscopic otologists favor this approach.(5)

Despite the advantages, concerns exist regarding the ultimate hearing results and complications from lifting the tympanic membrane from the malleus. Lifting the tympanic membrane requires manipulation of the ossicles, which theoretically could cause sensorineural hearing loss.(6) Conductively hearing loss is also possible if the tympanic membrane does not heal appropriately and the neotympanum lateralizes or has anterior blunting.(7) Additionally, the tympanic membrane is densely adherent to the malleus and residual squamous tissue could result in cholesteatoma.

In previous study, we detailed the microscopic over-under cartilage tympanoplasty approach using a case series from one of our coauthors (M.A.A.).(8) The senior author for the present study (R.M.) is an endoscopic otologist who performs both 1) the over-under technique, where the tympanic membrane is elevated from the malleus, and 2) the underlay cartilage technique, where the tympanic membrane is left on the malleus. This allowed us an opportunity to compare over-under and underlay techniques in the endoscopic setting, and to evaluate whether dissecting the tympanic membrane from the malleus had any clinical impact on hearing or complications, using a separate dataset. We hypothesized that the hearing results were similar between over-under and underlay techniques, so equivalence testing was used.

## MATERIALS AND METHODS

The authors assert that all procedures contributing to this study comply with the ethical standards of the relevant national and local institutional guidelines on human experimentation and with the Helsinki Declaration of 1975, as revised in 2008. This study was approved by our local institutional review board (IRB number 19-082-OLOL).

### Patient Selection

In this retrospective cohort, cases of endoscopic over-under cartilage tympanoplasty were identified at 1 institution between January 2015 and January 2019. They were age and gender matched to a control of underlay cartilage tympanoplasties performed during the same period. Cases were excluded if they had preoperative or intraoperative cholesteatoma or chronic otomastoiditis, if a mastoidectomy or ossiculoplasty was performed, or if postoperative audiograms were not available.

### Operative Intervention

**FIG. 1.**
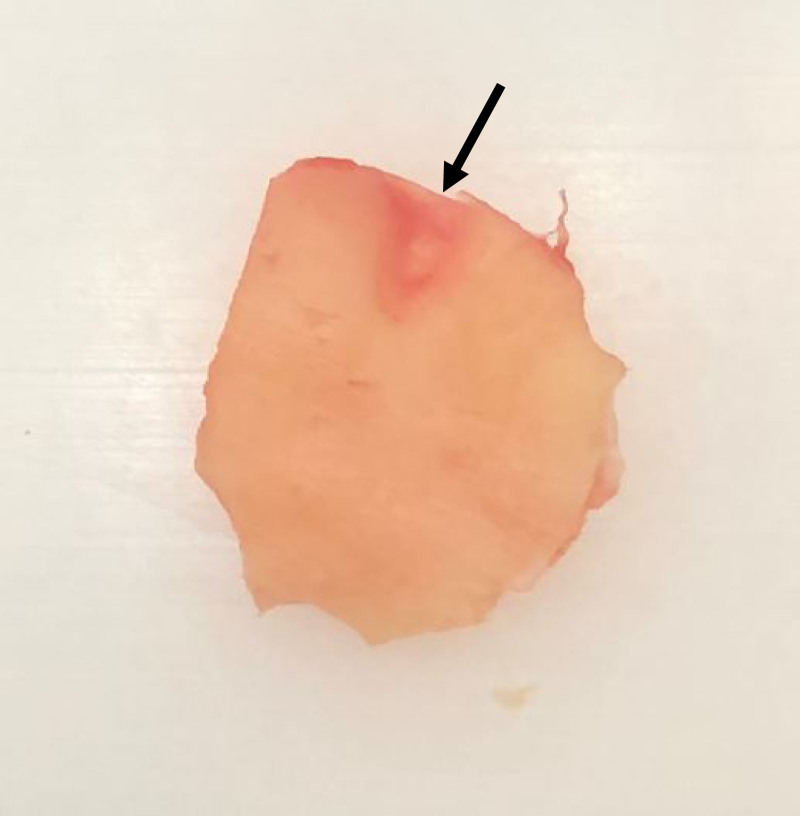
A cartilage-perichondrium island graft prepared for an over under tympanoplasty. Perichondrium has been removed from one side, and a groove (arrow) was cut in the cartilage for placement on the malleus. A similar graft was used for the underlay, fashioned with a smaller cartilage island, and without the groove superiorly.

**FIG. 2.**
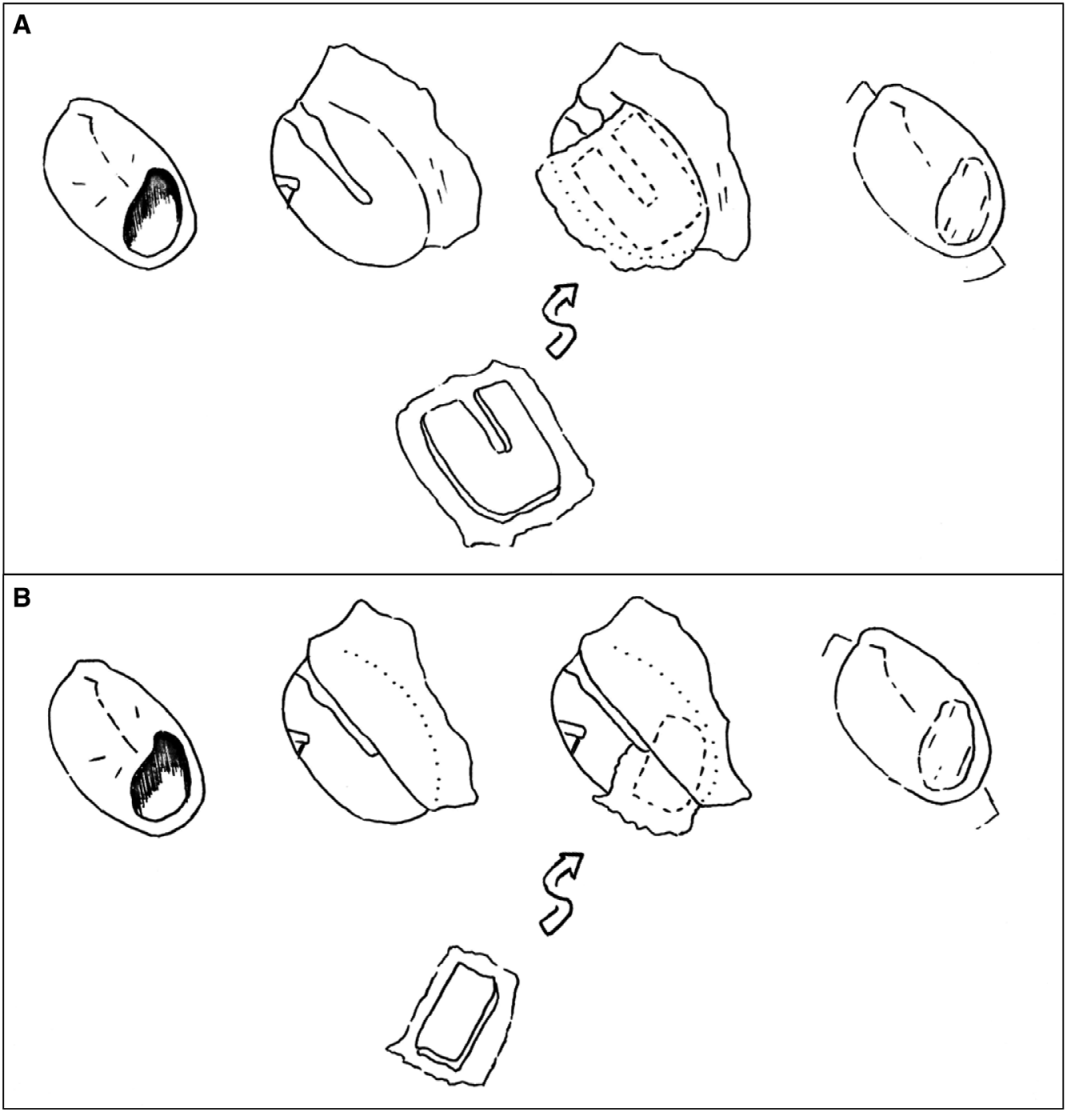
Placement of the cartilage graft. The over under cartilage (*A*) is placed medial to the annulus and lateral to the malleus. The underlay graft (*B*) is placed medial to both the annulus and the malleus. For both, the cartilage side of the cartilage-perichondrium island graft faces medially when placed in the ear.

Briefly, the key characteristics of the over-under tympanoplasty were that the tympanic membrane was elevated from the malleus and the cartilage island graft obtained from the tragus or concha (Fig. 1) was placed lateral to the malleus and medial to the annulus (Fig. 2A). A groove in the cartilage graft was fashioned to fit over the malleus. Using an argon laser at 1500 mW for 200 ms per fire, any remaining squamous tissue was removed from the handle of the malleus. The technique used was a minor modification of that presented in Erbele et al.(8) to accommodate use of the endoscope. In contrast to that study, a postauricular incision was not made and an anterior canalplasty was not performed.

In cases where an underlay tympanoplasty was performed, a cartilage island graft without a groove for the malleus was placed medial to both the malleus and the annulus (Fig. 2B). The tympanic membrane was not elevated from the malleus in these cases.

The intraoperative decision for performing an over-under tympanoplasty instead of an underlay was made if the malleus was retracted medially and there was limited space to perform an underlay graft, or if the location of the perforation made an underlay graft more challenging—particularly if it was in the anterior-superior quadrant. Also, in cases where tympanoplasty was performed for a flaccid tympanic membrane, the over-under technique was preferred.

All cases were performed with 0° and 30° 3-mm endoscopes, 14 cm in length.

### Data Collected

Pre- and postoperative audiograms were evaluated. Preoperative audiograms were typically obtained within 1 month before the procedure. For consistency, the first postoperative audiogram was used for analysis, typically at the 3-month point. Thresholds for air and bone conduction were obtained at 500, 1000, 2000, 3000, and 4000 Hz; 250, 6000, and 8000 Hz were additional obtained for air conduction. Thresholds at 3000 and 6000 Hz were interpolated, if needed.(9) Pure-tone averages (PTA) were determined by averaging air conduction at 500, 1000, 2000, and 3000 Hz, as recommended by the American Academy of Otolaryngology.(10) Bone PTA were also obtained at the same frequencies. Word recognition scores were obtained. Qualitative descriptions regarding the size and location of the perforations were obtained from the clinic and operative encounters; quantitative data were not available at the time of this retrospective review.

All cases were evaluated for complications by reviewing all available postoperative neurotology and otolaryngology notes. Postoperative perforation, cholesteatoma, external auditory canal scarring, and donor site morbidity were specifically assessed.

### Data Analysis

Data was analyzed in R version 3.5.3 (R Foundation for Statistical Computing, Vienna, Austria) within RStudio version 1.1.463 (RStudio, Inc., Boston), along with the additional R packages “tidyverse” version 1.2.1, (11) “psych” version 1.8.12, (12) and “TOSTER” version 0.3.4.(13) Paired *t* tests were used for both intergroup and intragroup changes in PTA. Fisher exact test was used to assess categorical data. A Wilcoxon rank sum test was performed on follow-up length. Equivalence testing for continuous data was performed on hearing results with “two one-sided *t*-tests”.(14) A difference of 3 dB was considered equivalent for air and bone conduction, based on evidence that this is the just noticeable difference in signal-to-noise ratio in PTA.(15) A difference of 4% was considered equivalent for word recognition score, allowing for variance in test-retest reliability.(16) Code for plotting audiograms was modified from previous study.(17,18) The scattergram plots were created with assistance of the Stanford web-based tool.(19)

## RESULTS

A total of 26 over-under endoscopic cartilage tympanoplasties were identified with audiograms available. These were age and gender matched to 26 underlay endoscopic cartilage tympanoplasties. There were 14 female patients and 12 male patients in each group. The median age for the over-under group was 15.5 years (range 6-71), and the median age for the underlay group was 14.5 (range 6-66).

The median follow-up length for the over-under group was 355 days (interquartile range [IQR], 140-564). For the underlay group, the median follow-up was 139 days (IQR, 104-402). Patients were routinely followed up at 3 and 12 months after surgery, and slightly more patients followed up at the 12-month point or longer in the over-under group. The difference in follow-up time was not statistically significant (*P* = 0.22).

Qualitatively, there were similar sizes of perforation for both groups, and anterior perforations were treated with both techniques. The over-under technique was exclusively used for anterior-superior perforations, however. Medial rotation of the malleus was often the key difference in technique selection, favoring the over-under technique in those cases. In cases where the tympanic membrane was intact but atrophic, the over-under technique was preferred.

**FIG. 3.**
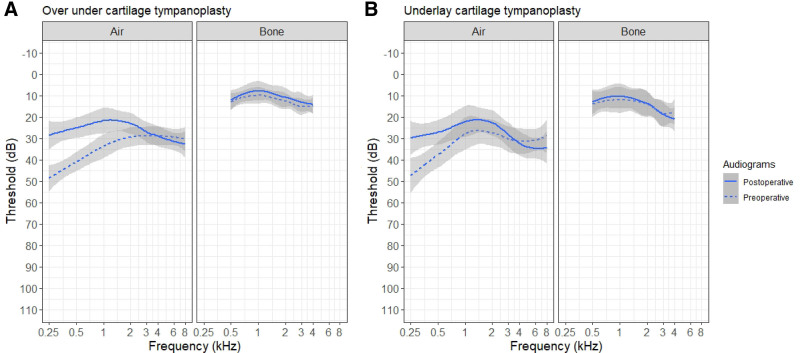
Averaged audiograms. Both the over under cartilage tympanoplasty (*A*) and underlay graft tympanoplasty (*B*) demonstrated improvement of air conduction, particularly at the lower frequencies.

**FIG. 4.**
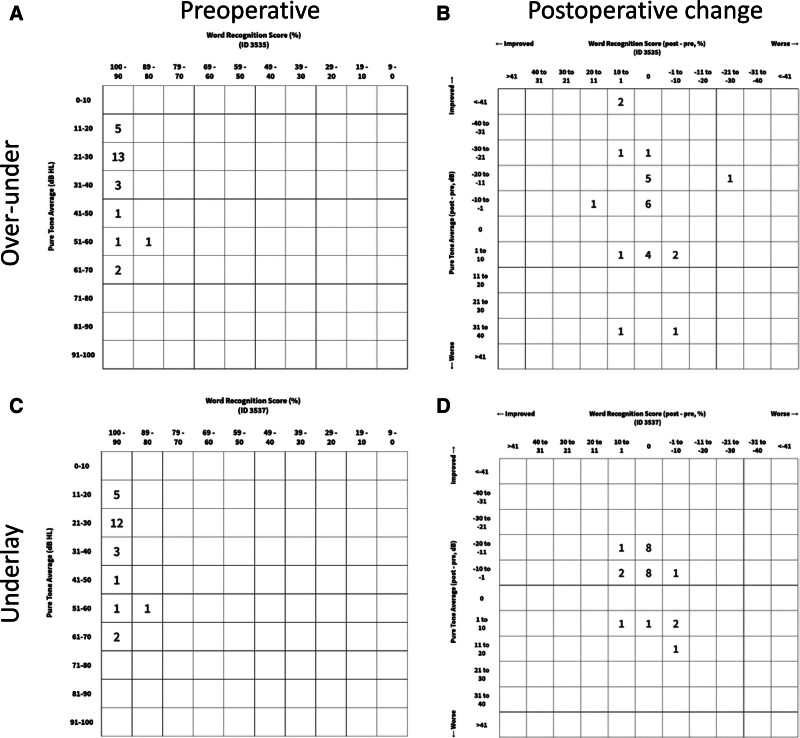
Scattergram plots. Over under graft (*A*) and (*B*). Underlay graft (*C*) and (*D*).

The changes in hearing results are summarized in Table 1. Averaged audiograms for the 2 groups are shown in Figure 3, and scattergram plots are shown in Figure 4. The improvement in air-conduction PTA was 9 dB for the over-under group and 6 dB for the underlay group. This improvement was statistically significant in both groups (*P* < 0.001 and *P* < 0.01, respectively), though the hearing results crossed into statistical equivalence bounds for the underlay group. The improvement in air conduction in the over-under group was noninferior to the underlay group (*P* < 0.05). Bone conduction did not worsen in either group. Word recognition score was unchanged after surgery and equivalent between groups.

**TABLE 1.** Hearing and equivalence testing outcomes

	Improvement from preop (mean, 95% CI)	Difference from preop	Equivalence from preop	Difference in technique	Equivalence in technique
Air PTA					
Over under (n = 26)	9 dB (5–14)	*P* < 0.001	Nonequivalent (postop superior)	*P* = 0.78	OU noninferior, *P* < 0.05
Underlay (n = 26)	6 dB (2–9)	*P* < 0.01	Postop noninferior, *P* < 0.0001		
Bone PTA					
Over under (n = 26)	2 dB (−1 to +4)	*P* = 0.17	Postop noninferior, *P* < 0.001	*P* = 0.49	OU noninferior, *P* < 0.01
Underlay (n = 26)	1 dB (−1 to +3)	*P* = 0.54	Postop equivalent, *P* < 0.05		
ABG					
Over under (n = 26)	7 dB (3–12)	*P* < 0.01	Postop noninferior, *P* < 0.0001	*P* = 0.42	OU noninferior, *P* < 0.05
Underlay (n = 26)	5 dB (2–9)	*P* < 0.01	Postop noninferior, *P* < 0.001		
WRS change					
Over under (n = 26)	–1% (–4 to +1)	*P* = 0.24	Postop equivalent, p<0.05	*P* = 0.25	Equivalent, *P* < 0.05
Underlay (n = 25)	0% (–1 to +2)	*P* = 0.81	Postop equivalent, *P* < 0.00001		

PTA across 500, 1000, 2000, and 3000 Hz. ABG, averaged across the same frequencies. Note that 1 patient did not have a postoperative WRS available.

ABG indicates air-bone gap; CI, confidence interval; dB, decibel; OU, over under tympanoplasty; PTA, pure-tone average; WRS, word recognition score.

There were no perforations in the over-under group and 3 in the underlay group, but this difference was not statistically significant (*P* = 0.24). No other complication required additional surgery, but there was 1 tragal keloid in the over-under group and 1 case of external auditory canal scarring in the underlay group. There were no cases of postoperative cholesteatoma in either group.

## DISCUSSION

Improving cartilage tympanoplasty has been an area of active research. Here we show that the endoscopic over-under cartilage tympanoplasty technique has equal or better hearing outcomes to the endoscopic underlay technique, and that bone conduction does not worsen after careful manipulation of the ossicles. Notably, there were no cases of cholesteatoma, lateralization, or anterior blunting in the over-under group. Finally, we show that perforation is rare for the endoscopic over-under technique. Taken together, this suggests that lifting the tympanic membrane from the malleus, when applied appropriately, does not cause untoward effects.

Recently, cartilage tympanoplasty has been compared with fascia, (2,20–36) acellular dermal matrix, (37) perichondrium, (38) and other cartilage techniques.(24,36,39–41) These comparisons rarely find statistically significant differences in hearing, but authors rarely perform statistical testing to evaluate whether the results are clinically similar.(42) Here we use equivalence testing, which allows evaluation of clinical similarity, and based the 3 dB smallest effect size of interest on what a patient might notice in real world hearing scenarios.(15)

It can be useful to show similar results in similar techniques when there are unique advantages to using one technique over another. Underlay cartilage tympanoplasty is an attractive technique when the perforation does not involve the anterior-superior quadrant and there is sufficient space in the middle ear to place a cartilage graft medial to the malleus. On the other hand, elevating the tympanic membrane from the malleus, as is performed in the over-under technique, allows access to repair of the anterior-superior quadrant and creates the opportunity to place a cartilage graft when the malleus is medially rotated and middle ear space is limited.(1,20,22,28,30,41,43–47) Demonstrating equivalent or better hearing results for the over-under technique supports its application in these more challenging perforations.

We also feel that this study reinforces previously published studies on the use of over-under tympanoplasty compared with underlays. Two recent articles have evaluated this, (1,47) and while neither used cartilage, reported high frequencies, or had a matched cohort intended to evaluate equivalences, both found favorable outcomes using the over-under approach.

Every attempt was made to control for confounders. All cases were performed by 1 surgeon at 1 institution, and all cases were performed with endoscopy. Cases were age and gender matched. Unfortunately, because the decision for over-under cartilage tympanoplasty was made intraoperatively to address more challenging perforations, these cases could not be matched for size and location of perforation. It is possible that this intraoperative decision introduced selection bias, but it is difficult to make the conclusion that this was a clinically important effect since the ultimate postoperative air-bone gap was equivalent or better in the over-under group compared with the underlay group. There was a difference in the shape of the grafts placed in each group, and this may have modestly affected hearing outcome. This study could have been improved with a longer follow-up of the control group and a larger sample size. This is also a retrospective study with its inherent limitations—particularly the fact that qualitative data was relied on for the size and location of the tympanic membrane perforations. Despite these limitations, the appropriate application of equivalence testing adds a unique perspective to the tympanoplasty literature.

## CONCLUSIONS

Hearing results for over-under endoscopic cartilage tympanoplasty are noninferior to underlay endoscopic cartilage tympanoplasty. This study supports the conclusion that lifting the tympanic membrane from the malleus is a reasonable and safe tool to address the anterior tympanic membrane and mesotympanum.

## FUNDING SOURCES

None declared.

## CONFLICT OF INTEREST

Book royalties, Elsevier (M.A.A.).

## DATA AVAILABILITY STATEMENT

The data that support the findings of this study are available from the corresponding author (R.M.) upon reasonable request.
